# Caregiver accompaniment and non-abandonment in voluntary assisted dying: Phenomenological analysis

**DOI:** 10.1017/S1478951525000501

**Published:** 2025-05-22

**Authors:** Natasha Michael, Jeanette Liebelt, Xavier Symons, David Kissane

**Affiliations:** 1 School of Medicine, University of Notre Dame, Sydney, NSW, Australia; 2 Faculty of Medicine, Nursing and Health Sciences, Monash University, Clayton, Melbourne, VIC, Australia; 3 Plunkett Centre for Ethics, Australian Catholic University, Darlinghurst, NSW, Australia; 4 Human Flourishing Program in the Institute for Quantitative Social Science, Havard University, Cambridge, MA, USA

**Keywords:** Euthanasia, physician-assisted suicide, palliative care, caregivers, qualitative research

## Abstract

**Objectives:**

Caregivers play a significant role in the process of Voluntary Assisted Dying (VAD), reporting stances of support, opposition, or ambivalence. Though caregiver vulnerability is recognized, little is understood about how caregivers adjust when patients seek VAD. We sought to appreciate how bereaved caregivers of patients in organizations that did not participate in VAD processed and adapted to the challenges faced.

**Methods:**

We purposefully recruited caregivers from cases reviewed in a retrospective study exploring how VAD impacted the quality of palliative care. We further expanded sampling to maximize diverse views. We used qualitative interpretative phenomenological analysis to explore unique caregiver perspectives.

**Results:**

Twenty-three caregivers completed interviews. Most were female, Australian-born, retired, identified with no religion, bereaved for 1–3 years, and in a caregiving role for 1–5 years. Caregivers sought accompaniment and non-abandonment across all stages of VAD. Coping was enhanced through framing and reframing thought processes and reconciling values. Caregivers bore responsibility through heightened emotions and experienced isolation and anticipatory grief as they reconciled perceived societal attitudes. Caregivers additionally failed to understand the rationale behind organizational stances and were unable to articulate the moral conflicts that arose. Impartiality from professionals was valued for caregivers to sustain care for the patient.

**Significance of Results:**

Despite feelings of vulnerability and isolation, caregivers demonstrated benevolence, courage, and self-compassion, reframing and accommodating their concerns. Professional accompaniment and non-abandonment necessitate solidarity and empowerment, without necessarily enabling VAD. Findings demonstrated the need for individuals and organizations to clearly articulate their willingness to continue to accompany patients, regardless of their position on VAD.

## Introduction

Caregiving is a selfless act endured by many involved in supporting patients with a life-limiting illness. Informal caregivers in palliative care sustain patients through challenging treatments, fostering hope through unpredictable trajectories, increased dependency, vacillating symptoms, and loss of identity. In doing so, caregivers sacrifice personal ambitions and endure emotional isolation and financial hardships (Alam et al. 2020; Gardiner et al. 2020; Gray et al. 2020). The ambiguity around treatment options and prognosis and their impact on outcomes add to caregiver burden (Loh et al. 2021; Wang et al. 2018). The caregiver burden is alleviated when caregivers are supported and included in the decision-making triad with patients and clinicians, aiming for concordance in decision-making and communication (Michael et al. 2022). Notwithstanding this, caregivers across international jurisdictions now face additional ethical and decision conundrums as palliative care patients consider the option of assisted dying (Goldberg et al. 2021; Scheeres-Feitsma et al. 2023; Thangarasa et al. 2022; White et al. 2023a).

Voluntary Assisted Dying (VAD) was introduced in Victoria, Australia, in 2017 and in Western Australia in 2019 (it is now available across states and territories). It allows adults aged ≥ 18 with a life-limiting illness and a prognosis of ≤ 6 months (≤12 months for progressive neurological diseases) to consider assisted dying through the use of self or clinician-administered substances following assessment by two clinicians and permit provision.(End of Life Law in Australia 2024) The VAD substance for self-administration is delivered by a statewide pharmacy service to the patient’s home or place of care, where patients and caregivers are instructed on procedures for self-administration. The legislations allow for individual and organizational conscientious objection with policy recommendations for any organization to manage their position and models of participation through transferring patients to alternative facilities or providing a locked cabinet for patients to self-access medication when an inpatient. Since the enactment of legislation and up to June 2023, 1167 Victorians and Western Australians have died following administration of the VAD substance, with the support of participating health practitioners and assumed support from their caregivers (VAD Board Western Australia Annual Report 2022-23; Safer Care Victoria Annual Report 2023-24).

A review of the impact of VAD on informal caregivers of patients with advanced illness described varying caregiver positions from supportive to ambivalence to opposition (Ganzini et al. 2006; Goldberg et al. 2021; Okishiro et al. 2009). Younger caregivers and those reporting lower levels of religiosity were more likely to articulate a sympathetic stance (Ganzini et al. 2006; Okishiro et al. 2009) but less than 20% of those endorsing VAD were willing to be directly involved (Emanuel et al. 2000). Studies of bereaved caregivers of patients who have availed of assisted dying have shown a myriad of challenges faced from variation in VAD processes across jurisdictions, competing values, moral and ethical dilemmas, fear of social stigmatization, relational strain, complex grief, post-traumatic stress and varied perceptions of the quality of end-of-life care (Gamondi et al. 2015; Georges et al. 2007; Goldberg et al. 2021). Nonetheless, caregivers have been found to play a significant role in facilitating VAD, describing positive outcomes such as satisfaction achieved in supporting a desired patient outcome and providing emotional and instrumental support, as well as negative consequences due to inadequate preparation and the burden of responsibility (Gamondi et al. 2018; Hashemi et al. 2023; Lowers et al. 2020).

Despite expanding legislation, an ongoing contention for caregivers is access and support through the VAD process (White et al. 2023b). Research suggests that palliative care and VAD services undertake models of cooperation, integration, or separation (Gerson et al. 2023, 2020). The challenge is accentuated for the ambivalent patient, simultaneously contemplating a natural death and VAD, with or without the involvement of the family (Michael et al. 2024). Caregiver isolation occurs with the desire for privacy, fear of stigmatization, and conflicting values, risking family dysfunction (Gamondi et al. 2018; Lowers et al. 2020). In this study, we aimed to specifically understand the experience of caregivers of palliative care patients in organizations that did not directly provide VAD through an interpretative phenomenological approach. We specifically sought to comprehend how caregivers processed and adapted to the many challenges faced.

## Methods

### Study design

**Figure 1.**
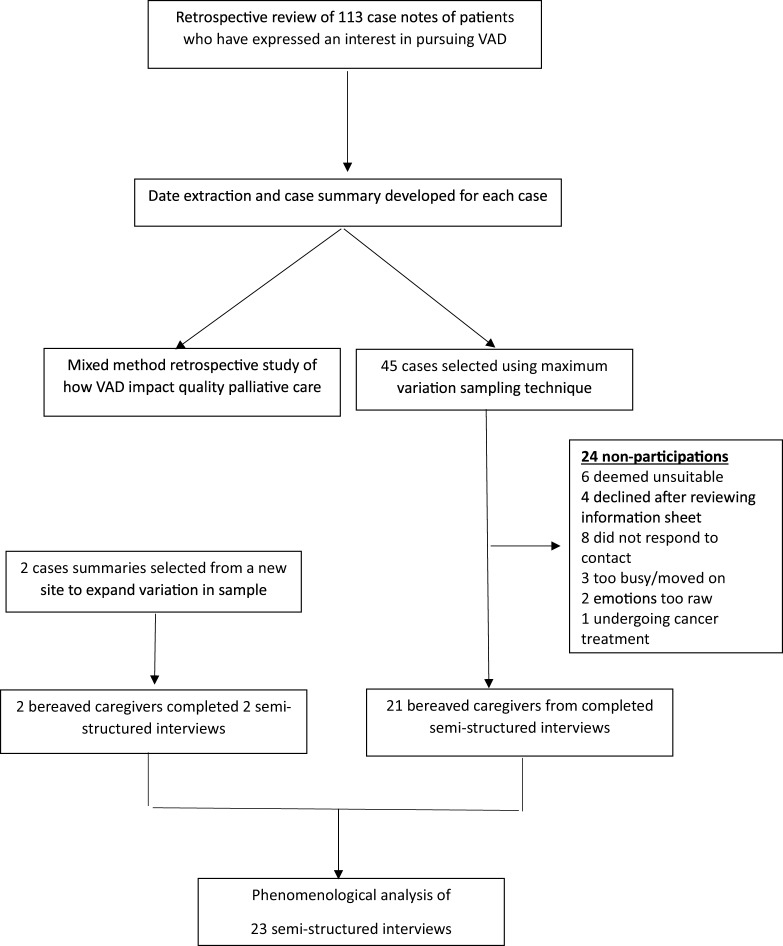
Study schema.

This study was a sub-study of a larger retrospective mixed method study, which reviewed 141 case notes of a consecutive cohort of patients who had expressed an interest in and subsequently pursued or did not pursue VAD (Figure 1) (Michael et al. 2024). Interpretive phenomenological analysis was used as it allows for a focus on how people make meaning of their lived experiences (Tuffour 2017). This approach to analysis occurs without the use of theoretical frameworks or prior assumptions, thus avoiding the influence of the authors’ preconceptions as much as possible. Ethics approval was obtained from the Human Research Ethics Committee of each participating site (01-04-08-22; 22081801; 2022-008;1988; 1988) and the trial was registered with the Australian and New Zealand Clinical Trials Registry (ACTRN12622001357741). Study reporting was guided by the Consolidated Criteria for Reporting Qualitative Research (Dossett et al. 2021).

### Setting, participants, and recruitment

Participants were recruited from three sites in Victoria and one in Western Australia. These included a service providing palliative care in a 22-bed specialist unit, consult and community services; a subacute service with a 32-bed specialist unit and community service; an acute hospital service with a 12-bed specialist unit, consult and community service, and a large metropolitan hospital with a 12-bed specialist unit and consulting services. Each site maintained a database of adult patients who had inquired about VAD; all patients would have had contact with a palliative care service.

A female researcher (JL), an experienced palliative care nurse with no prior contact with participants, examined the deidentified case summaries from the retrospective study (Michael et al. 2024) and used a purposive maximum variation sampling technique to identify cases that could provide unique perspectives (Moser and Korstjens 2018). Selected caregivers were deemed eligible for the study if the patient’s medical records confirmed that the caregiver was aware of the patient’s exploration of VAD and contact details. JL contacted site investigators to confirm the suitability of selected cases. The site investigators approached the primary clinicians involved with the patient and asked to contact the bereaved caregiver by telephone to explore interest in interview participation.

Interested participants were contacted by JL via telephone or email and provided with a participant information sheet about the study. Subsequent agreement to participate implied consent and was recorded. Interviews were conducted over the telephone and audio recorded by JL, an experienced interviewer, using an interview guide prepared by the research team based on findings from a previous study and published literature (Table 1) (Goldberg et al. 2021; Michael et al. 2024; Thangarasa et al. 2022; White et al. 2023a). Participants were all present at home, participated alone, and field notes were maintained. Interviews were audio-recorded, anonymized, and transcribed verbatim. Due to the sensitive nature of the inquiry, content validation was not sought. Interviews continued until data saturation was achieved. Completed transcriptions were imported into qualitative data management software.

**Table 1.** Questions for bereaved caregivers

Questions	Prompts
What factors were associated with why the person you cared for may have inquired about or sought VAD?	Did you share some of their concerns and views on accessing VAD?
	Were there any mixed feelings or ambivalence about the use of VAD? How were these worked through?
	Did this impact on your relationship with them?
	Did their interest in pursuing VAD affect their subsequent care or the care of your family?
Reflecting on the overall experience of accessing VAD, were there any challenges faced?	How did the VAD process affect you, the person you cared for, the team caring for them, and your family?
	What caused you or the person you cared for distress?
	Were there any specific ethical challenges that you faced?
Thinking about the dying process, did that evolve smoothly?	Did the person you care for die naturally or with the use of the VAD substance?
	Were there any moments of concern?
	Did you feel supported through the process?
Grieving is a very personal experience. How was your grieving impacted as the person you cared for considered or accessed VAD?	How has your grief changed since the loss?
	Do you think VAD has impacted in some way on your grief and that of your family?
What was your experience of the palliative care received by the person you cared for and yourself?	Were there any challenges accessing/receiving health care and palliative care services while the person you cared for was considered VAD?
	Did the involvement with VAD affect the quality of palliative care you and the person you cared for experienced?
	What do Palliative Care Services need to consider when someone inquires about or seeks the provision of VAD?

### Data analysis

The initial analysis was conducted by JL and NM, who read the deidentified interview transcripts independently and familiarised themselves with the content of the responses. Significant statements were identified, formulated meanings were derived, organized into formulated concepts, and subsequently clustered into subthemes. JL and NM compared and reconciled concepts and subthemes to improve methodological rigor. DK reviewed developing concepts and subthemes, derived meanings, and assisted with discrepant views until a consensus was reached. A final overarching theme was developed through the integration of each subtheme into textural descriptions.

## Results

A total of 30 potential caregivers were identified, of whom 23 caregivers of 18 patients completed semi-structured interviews (Figure 1). Caregiver sociodemographic details are summarised in Table 2. The majority were female, Australian-born, retired, and identified with no religion. Most had spent between 1 and 5 years caring for a partner/spouse or parent and had been bereaved for between 1 and 3 years. The mean interview time was 52:54 min.

**Table 2.** Bereaved caregiver and corresponding patient characteristics

Characteristics	Caregivers *N* = 23 (%)	Corresponding patients *N* = 18 (%)
Age in years, mean (SD), (range)		
Sex		
Female	12 (52.2)	11 (61.1)
Male	11 (47.8)	7 (38.9)
Patient diagnosis		
Gastrointestinal		10 (55.6)
Breast		2 (11.1)
Other		6 (33.3)
Country of birth		
Australia	19 (82.6)	13 (72.2)
Other	4 (17.4)	5 (27.8)
Religion		
No religion	14 (60.9)	9 (50.0)
Christian	7 (30.4)	4 (22.2)
Other	2 (8.7)	1 (5.6)
Unknown		4 (22.2)
Employment status		
Employed andworking	8 (34.8)	
Unemployed, not seeking work	2 (8.7)	
Retired	13 (56.5)	
Length of time as a caregiver		
<6 months	5 (21.7)	
6 months to1 year	6 (26.1)	
1–5 years	9 (39.1)	
>5 years	3 (13.1)	
Care-giver relationship to patient		
Partner/spouse	11 (47.8)	
Sibling	1 (4.4)	
Offspring	11 (47.8)	
Time since bereavement		
6–12 months	2 (8.7)	
1–3 years	18 (78.3)	
>3 years	3 (13.0)	

*Corresponding patient details were inaccessible for 2 patients, 1 patient was represented by 2 caregivers, and 1 patient was represented by 3 caregivers.

Our findings’ overarching theme for caregivers was seeking accompaniment and non-abandonment across all stages of VAD (Supplementary Material), which was supported by four subthemes that involved how to enhance coping, bear responsibility for the decision that was unfolding, reconcile with perceived societal attitudes and sustain care provision for their loved one.

### Subtheme A: Enhancing coping

**Figure 2.**
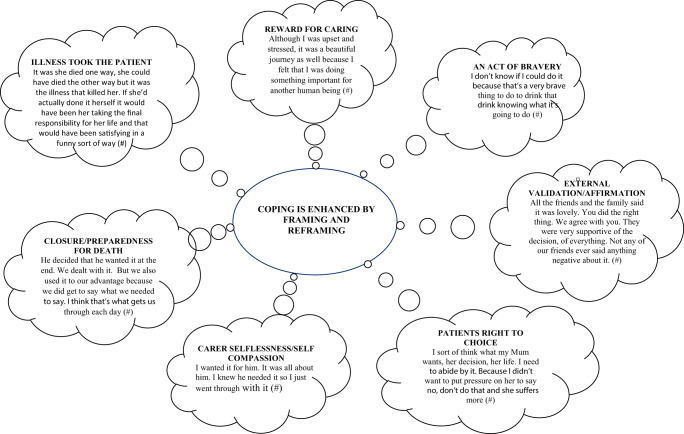
Caregiver framing and reframing.

*Framing and reframing*: Despite views contradictory to the patients, caregivers challenged their thought processes, framing and reframing concerns as they sought to accommodate them while supporting their loved ones (Figure 2). This challenge was enabled through affirmation or validation from friends, “I didn’t hear one person say anything about it” (#25); members of the clergy, “I went and talked to a priest about it” (#26); and health professionals, “I needed to speak with someone who was wise and knowledgeable (a psychologist)” (#13). The determination and certainty of the patient in pursuing the VAD path allowed for adjustment,

(Patient) showed no sign of concern whatsoever through all the steps which continued to reiterate to me that we were doing the right thing. (#1)

A strong feature was the caregiver’s motivation to meet the loved one’s wishes and a sense of satisfaction in achieving this.

The best thing that helps me whenever I think about it is just that she had that control and that’s what she wanted. So, it was a very good thing from that perspective. (#1)

*Reconciling values*: Participants who expressed spiritual or religious views articulated how they reconciled contradictions in values,

I don’t need anyone to confirm my faith. I gave her a lot of strength at times because I have a rock-solid faith. We both believe in a loving God and then also that we’ve been given free will and so the fact that (patient) was able to use her free will. (#13)

Another caregiver described her response to her husband’s desire for VAD, differed in their “I told him off about only thinking about himself” (#11), reminding the patient that there were “people who still value you being here” (#11). Others considered strategies to avoid conflict, with one describing “a pact” (#8), whereby,

when you walked into the room where (pt) was, we always laughed. No problem was made to be a big problem. Even though it was. But we never made him feel like it was an effort for us to do it. (#8)

### Subtheme B: Arduous responsibility

*Eliciting heightened emotions*: Caregiver demands were attenuated, as managing the patients’ symptoms was a “full time job” (#21) with a “spreadsheet of all the medications” (#21). One felt abandoned by the patient’s “doctor for over 20 years” (#9), as VAD was viewed as belonging to “the too-hard basket” (#9). Heightened caregiver anxiety was apparent, “we’re not trained. We didn’t know what we were doing” (#8),

I’m not a nurse. I found I was very tense about looking after him and being responsible for his pain relief, even with all the care that the visiting nurses provided. (#2)

Vulnerability and exhaustion were apparent as “the stakes are [were] high” (#20). The engagement with the statewide services that delivered the medication and the VAD doctor evoked mixed reactions. A caregiver shared that following the training for the self-administered medication,

I broke down and then I had to go to bed after that. And I woke up and thought I was having a heart attack. (#7)

Another described the relief at the support from the VAD doctor as they “did not want to be the person who would mix it [the medication] up…emotionally it would have been very difficult to do” (#21).

*Navigating VAD*: Trying to navigate the process of VAD with a deteriorating patient was described as “too long” (#13) and “a nightmare” (#9), with a caregiver stating that the “patient didn’t realize how many steps she had to go through” (#13). Particular challenges arose when the patient started declining, as caregivers found themselves in, “unknown territory because [pt] wasn’t able to make decisions” (#21). Caregivers struggled with the multiple stages of assessment, with one stating,

by the time you organize it, my Mum will be dead. (#9)

Many sought “services out there that can help navigate” (#9), expressing concern that the process of VAD was overly burdensome on the patient “when you are terminal and you’re sick and you don’t have the strength to do it” (#9). Nonetheless, caregivers demonstrated genuine benevolence and courage, persisting despite being “upset and stressed” (#1). One described a “beautiful journey” (#18) whilst another expressed self-compassion, stating

It was hard on me because I was not party to what was going to happen. So, I’ve sort of forgiven myself for that. (#13)

### Subtheme C: Companioned isolation

*Fear of judgment and internal conflicts*: VAD impacted relational care, with the fracturing of family cohesion and the loss of trustful bonds. A caregiver described the challenge of not supporting the patients’ decisions, stating, “For me it was difficult…… I didn’t really want (pt) to make the decision that she made, which I had to support it but it went against my religious view” (#13). These conflicts perpetuated emotional isolation,

It felt very isolating. And I know he felt morally judged in a way that was difficult to

rationalize because it’s such a personal decision. And it’s a decision you’re making at the most vulnerable point in your life. (#21)

This was compounded by the challenges of facing the reality of VAD,

You know, you can talk about it all you want…… but when it comes down to it. God, the dealing with the VAD stuff is the hardest bit for me. (#7)

*Impact on the grieving process* Anticipatory grief was apparent as a caregiver described. “Just sitting, watching him sitting in a chair……Evenings are full of distress, tears of sadness, confusion, frustration” (#2). Many caregivers “did not feel supported enough” (#21), having to “do all the stuff myself” (#9), outlining the impact,

I cracked it because I had been mixing up all the medication and I was so tired because I hadn’t had any sleep. I said, I can’t do this anymore. I was just breaking open. (#19)

Reflection formed part of the grieving process. A caregiver described the lingering angst that arose from having to support something they were morally uncomfortable with, “I was going along with it, but I was praying she would be healed” (#13) and its protracted impact “it was a difficult thing for me, and it did remain a difficult thing a year later” (#13). Another suggested that families required early and detailed preparation for the processes involved in VAD and dealing with the psychological strain as “this could really pull you apart” (#7). The gravity of the act attenuated grief,

because you’re giving something, you’re giving them a poison or whatever it is, you know, to kill them. (#3)

### Subtheme D: Conscientious objection and care provision

*Trust despite conflict*: Caregivers, who understood why some clinicians were conscientious objectors to VAD, still wished for the involvement of someone “who knew the patient and was involved in his care” (#21). Caregivers particularly valued those who held “a neutral perspective” (#1),

Just be accepting. To listen and reassure that they will still be there. (#2)

Professional behavior contradictory to this desire fostered feelings of abandonment, “the workers didn’t want to discuss it. They just sort of brushed it” (#9). Others articulated the benefit of a non-judgemental approach, describing the nurses as “fantastic” (#18) and

very clear about their boundaries [and] fitting in around us. (#13)

*Impartiality in care provision*: Whilst caregivers understood that an organization or individual may object to VAD, they sought conversations to be held in an “impartial way” (#1), stating “it does not matter which way we go…the two [views] should come together and work out a way that respects each other” (#18).

The ongoing provision of palliative care was paramount for many, where caregivers understood the stance of individual health practitioners, “I don’t believe they should be forced into doing something they don’t believe in” (#21) and sought solutions stating,

Maybe those Doctors don’t need to be involved in the actual event but I think the support in how to manage it or leading up to it could perhaps be considered helpful to the patient at the time. (#21)

*Religion blamed for moral objections*: Many lacked understanding as to why individuals or organizations held a particular stance toward VAD, seeking “transparency” and wanting to have it “up front with them” (#19), as honesty was deemed integral to trusting relationships. Caregivers appreciated the conflict of values, “we have a situation where you don’t agree because of your religious values which are understandable” (#18), but failed to understand the deeper rationale behind organizational stances and could not articulate the moral conflicts that arose for individuals. They wanted an “avenue to peace at the end of life” (#22), recognizing that it was “different for different people” (#22). One caregiver sought an understanding of,

different lives, different belief sets. Different recipes for peaceful death, which is just the ultimate gift to your family and loved ones as you say goodbye. (#18)

## Discussion

Our findings demonstrate caregiver vulnerability in VAD and the need for individuals and organizations to clearly articulate their willingness to continue to accompany patients through their illness despite non-participation in VAD. Caregiver coping was challenged by discordance of attitude about VAD with their loved one, requiring some means to understand an alternative viewpoint or reconcile differences in values. On the other hand, caregiving could be taxing, having to endure the unfamiliarity of the VAD process, feeling isolated despite being in the presence of family and friends, and living with the fear of judgment. Caregivers were relieved to relinquish the responsibility of administering the VAD substance to the doctor and adhere to patients’ wishes. Nonetheless, dedication to the act of caring was sustained, with selflessness and benevolence demonstrated. Caregivers sought accompaniment, transparency, and mutuality, regardless of their stance on VAD.

In a narrative synthesis of qualitative studies of caregivers at the end-of-life and assisted dying, Lowers et al. proposed a modified, stress-coping conceptual model of coping in the context of VAD (Hudson 2003; Lowers et al. 2020). They suggest that in VAD, the expectation of impending death and a shorter period of caregiving may ease the caregiver burden, but recognize that caregiver isolation and inadequate health care professional support are notable stressors (Lowers et al. 2020). Our findings suggest that caregivers sought to “make sense of” stressors using cognitive change strategies. In recognition of these stressors, Brown et al. argue for a model of patient and family-centered care in VAD, promoting interdisciplinary care throughout the illness, consistency of providers, anticipatory guidance, appropriate bereavement support, and emphasizing investment in relationships with caregivers (Brown et al. 2022). Nonetheless, services continue to fall short of meeting these recommendations, with caregivers clearly articulating the lack of preparation, therapeutic relationships, and bereavement support.

The process of caregiver accompaniment can be challenging when VAD is sought, yet provider conscientious objections require consideration beyond that of systemic solutions for rapprochement, as suggested by Peisah et al. (2023) or “reasonable accommodation” as suggested by White et al. (2023b). Our findings suggest that all phases of caregiving are affected by VAD, with caregivers articulating the physical pain of suffering, feeling “impotent” or “abandoned” (Levinas 1988). Thus, caregiver accompaniment seeks that one “walks with,” lending solidarity, counsel, empowering but not enabling VAD. Challenges of accompaniment may be perceived by practitioners or organizations objecting to VAD, who then seek boundaries of involvement. However, Hood-Patterson reminds us that the accompanier places front and center relational obligations in caring (Hood-Patterson and Carter 2023). But how equipped are palliative care practitioners to provide for the arduous work of staying present and empathetic toward caregivers in situations that may evoke distress? Is it possible that a shared vulnerability provides the path to staying present? Our data points to the need for an honest statement explaining the clinician’s commitment to ongoing caregiver support while standing aside from involvement in VAD as a personal moral stance and seeking agreement on such a model of care. Importantly, though, such a clinician ought to signal their willingness to discuss views about VAD whenever this could be helpful.

Our data further revealed the need to explore the caregiver’s emotional reaction, seeking to ascertain if they require independent support, how VAD affects family functioning, and how it creates anticipatory grief or concern. Creating a non-judgmental space where understanding the decision-making process and family communication is necessary for accompaniment. Exploring ambivalence about dying, reframing the value of life, reconciling values, and thus enhancing coping while educating about what palliative care can contribute is part of accompaniment and necessary to ensure ongoing caregiver care in bereavement.

## Strengths and limitations

The multisite nature of our study brings varied perspectives and highlights areas of caregiver support that individual services may need to focus on considering expanding legislation internationally. In our analysis, we did not distinguish if a patient eventually died of VAD or naturally. Our sample was fairly homogenous, representing predominantly female, Australian-born, retired, and non-religious participants, therefore limiting the generalizability of the results. The subjectivity of a qualitative phenomenological approach is unavoidable due to the researcher’s reflectivity. Future longitudinal studies measuring coping, bereavement outcomes, and family functioning following death by VAD may provide valuable information on long-term outcomes and their impact on individuals and health care services.

## Conclusion

The significance of unrecognized distress and the emotional toll on caregivers when caring for patients seeking VAD requires specific attention, future research, and considered interventions to prevent caregiver psychopathology, maladaptive coping, and ambivalence. Caregivers require solidarity, counsel, and empowerment through their caregiving journey. This requires a harnessing of their adaptive capabilities and selflessness in caregiving and the creation of a non-judgmental space and shared vulnerability to enhance coping. Concurrently, caregivers require education about what palliative care can contribute, regardless of a patient’s choice to pursue VAD.

## Supporting information

Michael et al. supplementary material Michael et al. supplementary material

## Supplementary material

The supplementary material for this article can be found at https://doi.org/10.1017/S1478951525000501.
