# Supplementary figures and images for: Caregiver accompaniment and non-abandonment in voluntary assisted dying: Phenomenological analysis
Source: Palliat Support Care. 2025 May 22;23:e105. doi: 10.1017/S1478951525000501 (PMC13166630; doi:10.1017/S1478951525000501)

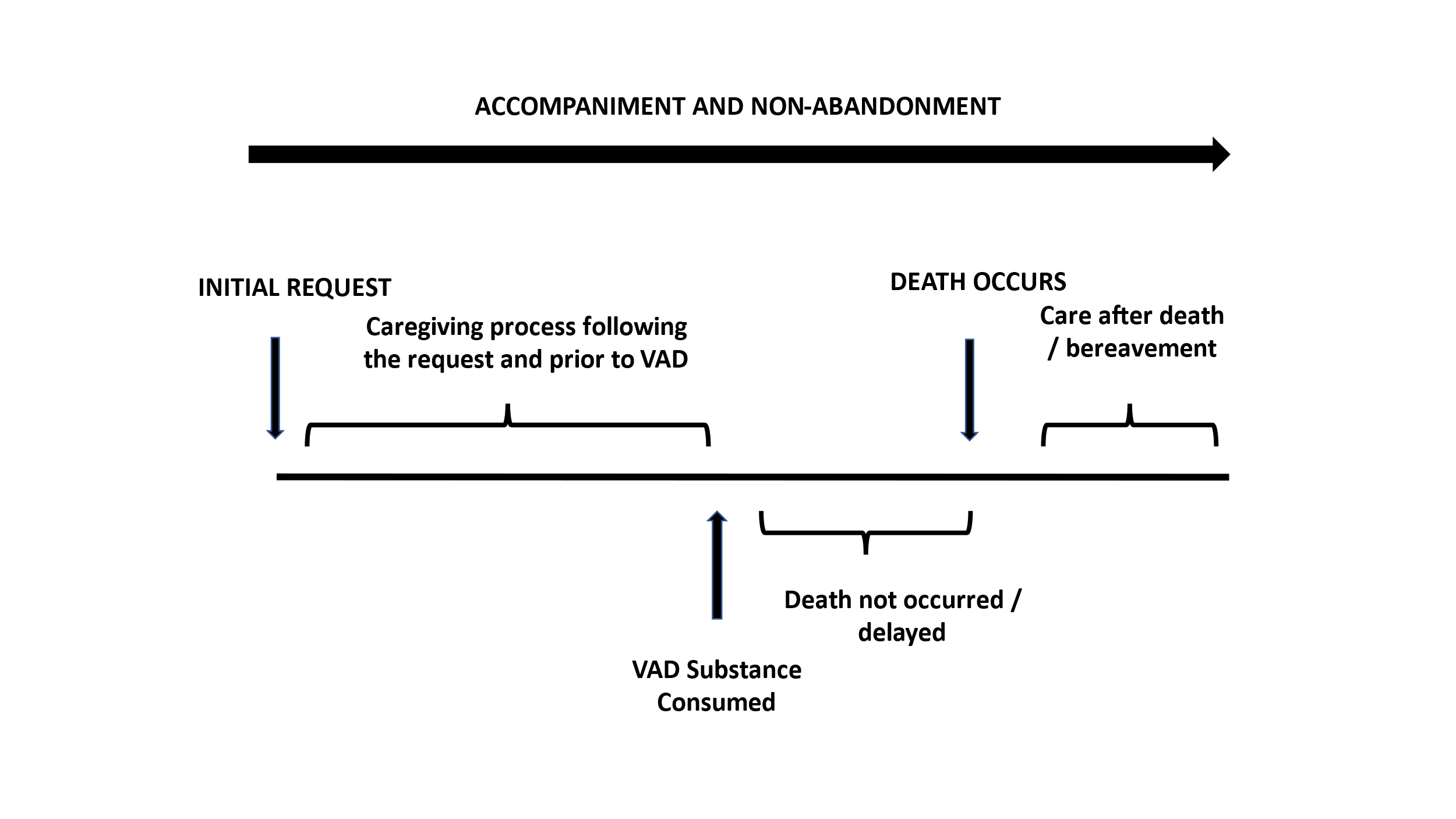

Supplement: Michael et al. supplementary material [file S1478951525000501sup001.docx]
